# An Experimental Investigation of the Risk of Triggering Geological Disasters by Injection under Shear Stress

**DOI:** 10.1038/srep38810

**Published:** 2016-12-08

**Authors:** Yixin Liu, Jiang Xu, Shoujian Peng

**Affiliations:** 1 State Key Laboratory of Coal Mine Disaster Dynamics and Control, Chongqing University, Chongqing 400044, China; 2 State and Local Joint Engineering Laboratory of Methane Drainage in Complex Coal Gas Seam, Chongqing University, Chongqing 400044, China

## Abstract

Fluid injection has been applied in many fields, such as hazardous waste deep well injection, forced circulation in geothermal fields, hydraulic fracturing, and CO_2_ geological storage. However, current research mainly focuses on geological data statistics and the dominating effects of pore pressure. There are only a few laboratory-conditioned studies on the role of drilling boreholes and the effect of injection pressure on the borehole wall. Through experimental phenomenology, this study examines the risk of triggering geological disasters by fluid injection under shear stress. We developed a new direct shear test apparatus, coupled Hydro-Mechanical (HM), to investigate mechanical property variations when an intact rock experienced step drilling borehole, fluid injection, and fluid pressure acting on the borehole and fracture wall. We tested the peak shear stress of sandstone under different experimental conditions, which showed that drilling borehole, water injection, and increased pore pressure led to the decrease in peak shear stress. Furthermore, as pore pressure increased, peak shear stress dispersion increased due to crack propagation irregularity. Because the peak shear stress changed during the fluid injection steps, we suggest that the risk of triggering geological disaster with injection under shear stress, pore, borehole, and fluid pressure should be considered.

In the early 1960 s, the relationship between hydraulic and mechanical processes in discontinuous rock masses received a lot of attention. Hydro-mechanical (HM) interaction^1^ were believed to have caused a series of events including dam failures, landslides, and injection-induced earthquakes. In recent years, fluid injection was applied to many fields, such as hazardous waste deep well injection^2^,^3^, geothermal field forced circulation^4^,^5^,^6^, hydraulic fracturing for oil and gas^7^,^8^, and CO_2_ geological storage^9^,^10^,^11^,^12^,^13^. However, researchers have observed that fluid injection affects seismicity. An example of injection-induced seismicity occurred at a deep well (3,638 m) for liquid waste injection at the Rocky Mountain Arsenal near Denver, Colorado^14^. Since the Denver Basin earthquakes, many injection-induced earthquakes have been documented^15^. Zoback and Gorelick^16^ suggested that fluid injection in deep wells can trigger earthquakes when the injection increased pore pressure is within the vicinity of preexisting potentially active faults. Gan and Frohlich^17^ suggested that gas injection may have contributed to triggering a sequence of earthquakes that have occurred since 2006 in and around the Cogdell field in Texas. Previous studies have examined hydro mechanical coupling during fluid injection^18^,^19^,^20^, but mainly focused on studying water-rock interaction and parameters such as pore pressure on rock mass^21^,^22^,^23^.

Drilling boreholes, injecting pressure acting on the borehole wall, and injecting pressure acting on the fracture wall will affect the stress distribution in the rock mass. Most studies have ignored the effects of drilling boreholes and injection pressure acting on the borehole wall, especially those under shear stress. Fletcher and Sykes^24^ suggested that mining would lead to greater stress differences; therefore, it would result in failure along appropriately oriented preexisting faults. Meier *et al*.^25^ performed a series of borehole breakout tests on Posidonia shale to study the influence of borehole diameter on borehole stability in unconventional black shale. In the study, a new apparatus was developed and employed that allowed direct shear tests to investigate injection-induced shear failure within rock masses. We studied the risk of triggered geological disasters of injection under shear stress.

## Results and Discussion

### Application to intact rock

The sandstone samples were obtained from the Three Gorges region in Chongqing, China at the upper Triassic Xujiahe Formation. The samples were fine terrigenous clastic sedimentary rocks, primarily composed of quartz, feldspar, chert, and muscovite with a grain size distribution of 0.1–0.5 mm. Drilling cores without obvious fractures were selected and cut into cubes with dimensions of approximately 100 × 100 × 100 mm. Under the condition of a borehole and injection pressure, the borehole with a 10 mm diameter and 60 mm depth was drilled into the center of the specimen to ensure the ideal shear failure surface through the center hole. Young’s modulus was 11.89 GPa, the Poisson ratio was 0.37, the uniaxial compressive strength was 55.97 MPa, and the density was 2.33 g/cm^3^.

To investigate the HM coupling characteristics of intact rock, direct shear experiments under three conditions were carried out. This included different pore pressures without borehole (NB), with borehole (WB), and injection water pressure (WP). Table 1 shows the experimental conditions and the three samples that were adopted for repeat experiments in each case. A constant shear displacement velocity of 0.1 mm/min was adopted during the experiments. These experiments measured shear displacement, shear stress, normal displacement, normal stress, injection water pressure, and flow rate.

#### Shear failure characteristics influenced by pore pressure

**Figure 1:**
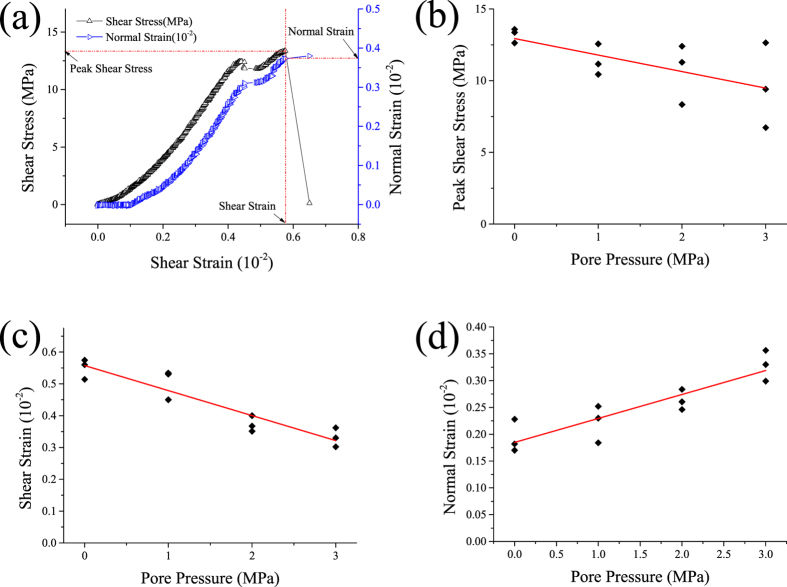
(**a**) Shear stress and normal strain vs. shear strain under a pore pressure of 0 MPa, (**b**) peak shear stress and (**c**) shear strain (**d**) normal strain of peak shear stress vs pore pressure.

Fig. 1(a) shows the curve of shear stress and normal strain vs. shear strain under a pore pressure of 0 MPa. Three parameters were employed to further investigation, including peak shear stress, shear strain, and the normal strain of peak shear stress. Fig. 1(b–d) displays the shear characteristic parameters of the specimens under different pore pressures. As shown in these figures, peak shear stress decreased as pore pressure increased (Fig. 1(b)). This was due to the mechanical effect of pore pressure in reducing strength and possibly to the chemical effect while reducing the friction coefficient between the particles and cementing material^26^. In addition, the peak shear stress dispersion simultaneously increased. When compression-shear stress impacted the rock specimen, tension cracks appeared and propagated^27^. Then the tension cracks were connected by shear cracks, which led to the final rupture^28^. Water was pressed into the tension cracks with pore pressure which changed the stress field of the crack tip and enhanced the tension crack to continue propagation^29^. Due to uncertainty of crack propagation, the length and aperture of tension crack and the effect by pore pressure are different. This can also be seen from Fig. 1(c–d); as pore pressure increased, the effective stress decreased and the normal strain increased. Compared with peak shear stress, the shear strain at peak shear stress decreased as pore pressure increased. It also remained consistent with tension crack propagation.

#### Shear failure characteristics influenced by drilling boreholes and injecting water

**Figure 2:**
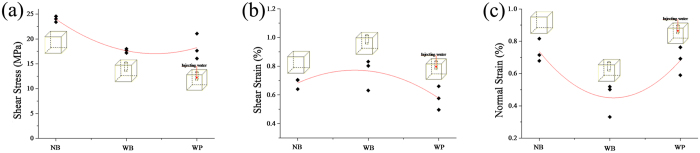
(**a**) Peak shear stress and (**b**) the shear strain and (**c**) normal strain of peak shear stress vs. different experimental conditions.

Fig. 2(a–c) displays the parameters at peak shear stress with different conditions, including specimens of intact rock (NB), with a borehole (WB), and injecting water into the borehole (WP). Peak shear stress decreased with the borehole condition, but it increased again after the water injection. Extensile fractures dominate the failure process when the applied normal load is very low^30^. The empty borehole provides space for deformation and may contribute to tension crack propagation, which decreases peak shear stress^26^. However, water injection prevented borehole deformation to a certain extent; it increased peak shear stress and the dispersion of them had a similar mechanism with different pore pressures. As mentioned above, the borehole provided more space for shear strain. Injecting water reduced shear strain through water penetration into the tension cracks (Fig. 2(b)) and promoted tension crack propagation instead of increasing shear strain. Similarly, drilling a borehole allowed for greater shear strain and avoided normal strain (Fig. 2(c)).

### Shear failure characteristics coupling HM influenced by normal stress

**Figure 3:**
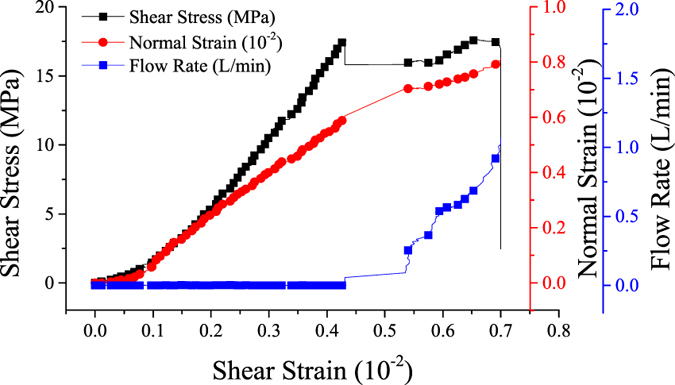
Shear stress and normal strain and flow rate vs. shear strain under a normal stress of 1.0 MPa.

**Figure 4:**
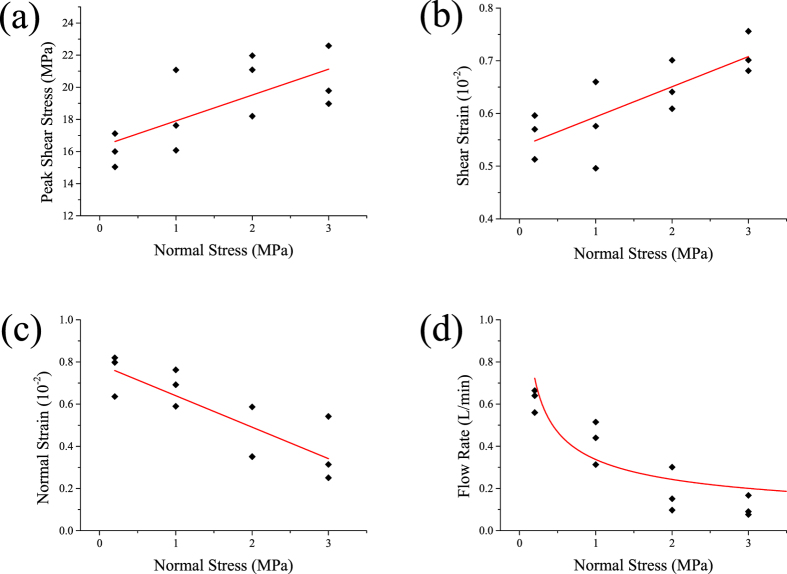
(**a**) Peak shear stress and (**b**) shear strain (**c**) normal strain and (**d**) flow rate of peak shear stress vs. normal stress.

To determine the mechanism of *in-situ* stress combined with injection pressure, direct shear tests were carried out. This involved different, but normal stress under a constant 1.0 MPa injection pressure. Figure 3 displays the corresponding changes of normal strain and flow rate of shear stress over shear strain under a 1.0 MPa normal stress. We can conclude from the flow rate changes that there was a crack between the borehole and specimen outer surface before shear stress peaked. The flow rate may be used as a parameter to describe the macro-crack coalesce process. Figure 4(a) shows that the peak shear stress increased consistently with the normal stress. Figure. 4(b–c) demonstrate how as normal stress increased, the normal strain decreased and shear strain increased. Figure 4(d) reflects the relationship between the flow rate and crack propagation. The flow rate curves at peak shear stress under different normal stress. The flow rate at peak shear stress decreased nonlinearly as normal stress increased. This indicated that as normal stress increased, the fracture aperture at peak shear stress decreased, as did the flow rate.

### Instrument development and methodology

**Figure 5:**
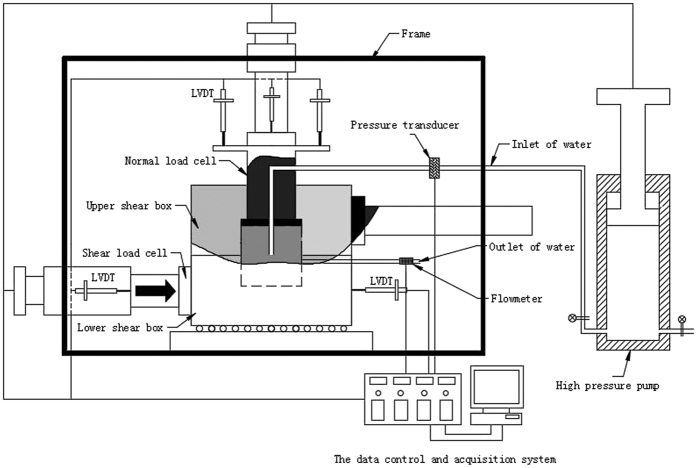
Sketch of the direct shear test apparatus.

A direct shear test apparatus for coupled HM was developed. It was designed to allow the testing of rock materials under different loading conditions, including a constant load or constant displacement rate. In addition to this, direct shear tests can be performed on intact rock specimens to investigate the hydro-mechanical characteristics influenced by drilling boreholes, injection pressure, and pore pressure. The test apparatus consisted of four components (Fig. 5): the shear box, the loading system, the measure system, and the data control and acquisition system.

### The shear boxes

**Figure 6:**
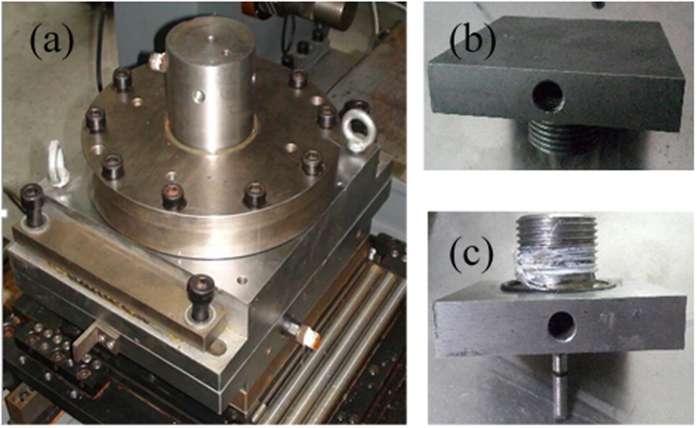
General view of the shear box. (**a**) normal pressure plate applied to (**b**) without borehole and (**c**) with borehole.

The shear boxes were built out of stainless steel (Fig. 6(a)) and an O-ring was employed to ensure that they were sealed. Shear stress was loaded on the lower shear box in the horizontal direction. Two plates were available to adapt the specimen with or without the borehole (Fig. 6(b–c)). With water injections through the inlet, the water flowed out through the cracks between the borehole and specimen’s outer surface that appeared. Pore pressure could be loaded by injecting water through the outlet and closing the water inlet. At the end of the experiment, the specimens could be removed by separating the upper and lower shear box.

### The loading system

The axial and horizontal loads were applied to the specimen by a servo-controlled electrical motor. The maximum loading capacity was 300kN (100 N precision) so that the constant displacement rate or constant load could be realized. The principle of fluid load was similar to that of the needle tube, which can maintain constant injection pressure or a water injection rate while simultaneously controlling the advancing speed or pushing pressure. The maximum loading capacity was 10 MPa (5kPa precision).

### The measuring system

A load cell integrated into the loading ram measured the axial and shear load. A water pressure transducer measured the fluid load and the water outlet flow rate was measured by the flow meter. Four and two LVDT were employed to separately measure the normal and shear displacement.

### The data acquisition and control system

The data acquisition and control system was a closed-loop. The computer received feedback signals from each transducer and was modified according to given commands. Data was saved in a.txt format, which can be easily managed for further analysis.

## Conclusion

We performed a new direct shear test apparatus to investigate HM characteristics. It allowed for the testing of rock materials under different loading conditions, including constant load or constant displacement rate. Two pressure plates were designed to adapt the specimen with or without a borehole. This new apparatus actualized a series of controlled direct shear tests.

Water was employed as a fluid medium to investigate the HM coupling characteristics of sandstone. Experiments were carried out to evaluate different parameters, such as drilling a borehole, injection pressure acting on the borehole wall, and injection pressure acting on the fracture wall. The pore pressure gradient field had a dominating effect on the propagation direction of the main tensile crack^31^, which decreased the peak shear stress and increased the normal strain. Different crack propagation paths led to different influences on peak shear stress.

Drilling a borehole provides space for deformation and may contribute to tension crack propagation and decreased peak shear stress. During water injection, water flowed through the cracks between the borehole and specimen outer surface before the shear stress peaked; the flow rate may be used as a parameter to describe the macro-crack coalesce process. As normal stress increased, the flow rate decreased nonlinearly and normal strain was restricted by normal stress.

In conclusion, injecting fluid underground, including drilling boreholes and fluid injection, will decrease shear stress and may increase the risk of triggering geological disasters.

## Additional Information

**How to cite this article:** Liu, Y. *et al*. An Experimental Investigation of the Risk of Triggering Geological Disasters by Injection under Shear Stress. *Sci. Rep.* **6**, 38810; doi: 10.1038/srep38810 (2016).

**Publisher's note:** Springer Nature remains neutral with regard to jurisdictional claims in published maps and institutional affiliations.

## Figures and Tables

**Table 1:** Experimental conditions.

Specimen no.		Normal Stress (MPa)	Pore pressure (MPa)	Borehole	Injection water pressure (MPa)	Water saturation coefficient (%)
NB	Case-1	3.0	0	No	0	0
	Case-2	3.0	0	No	0	100
	Case-3	3.0	1.0	No	0	100
	Case-4	3.0	2.0	No	0	100
	Case-5	3.0	3.0	No	0	100
WB		3.0	0	Yes	0	0
WP	Case-1	3.0	0	Yes	1.0	0
	Case-2	0.2	0	Yes	1.0	0
	Case-3	1.0	0	Yes	1.0	0
	Case-4	2.0	0	Yes	1.0	0
